# Development and computer-assisted validation of a radio frequency identification system for tracking individual chicken visits to functional areas

**DOI:** 10.1016/j.psj.2025.105627

**Published:** 2025-07-31

**Authors:** Serge Alindekon, T. Bas Rodenburg, Jan Langbein, Birger Puppe, Olaf Wilmsmeier, Sebastian Wille, Helen Louton

**Affiliations:** a Animal Health and Animal Welfare, Faculty of Agricultural and Environmental Sciences, University of Rostock, Justus-von-Liebig-Weg 6b, 18059 Rostock, Germany; b Animals in Science and Society, Faculty of Veterinary Medicine, Utrecht University, Yalelaan 2, 3584 CM, Utrecht, the Netherlands; c Behavior and Welfare, Research Institute for Farm Animal Biology (FBN), Wilhelm-Stahl-Allee 2, 18196 Dummerstorf, Germany; d Behavioral Sciences, Faculty of Agricultural and Environmental Sciences, University of Rostock, Justus-von-Liebig-Weg 7, 18059 Rostock, Germany; e Wilmsmeier Solutions, Hermann-Löns-Str. 108a, D-32547 Bad Oeynhausen, Germany; f Wille Engineering / Dipl.-Ing. Sebastian Wille, Volkerstraße 14, D-65795 Hattersheim am Main, Germany; g Chair of Animal Welfare, Ethology, Animal Hygiene and Animal Husbandry, Department of Veterinary Sciences, Faculty of Veterinary Medicine, LMU Munich, Veterinaerstr. 13/R, 80539 Munich, Germany

**Keywords:** RFID tracking, Automated behavioral tracking, ArUco markers, Camera detection, Technology validation, Precision livestock farming

## Abstract

Understanding how laying hens interact with functional resources—such as drinkers, feeders, perches, nest boxes, and wintergardens—is essential for meeting their physiological needs and enabling species-specific behaviors. This knowledge is crucial for poultry welfare assessments and precision livestock management. However, traditional ethological data collection methods, including direct observation and manual video analysis, are labor-intensive, prone to observer bias, and impractical for individual-level tracking. To overcome these challenges, we developed and validated an RFID-based system for automated, non-invasive tracking of individual hens' visits to key resources, using an established ArUco-based video annotation system as the reference standard. For validation, twenty-one laying hens were fitted with RFID leg bands and 3D-ArUco markers and monitored over five days in a mobile barn setup equipped with ultra-high-frequency RFID antennas. Alignment between data from the RFID and 3D-ArUco systems allowed calculation of performance metrics such as the F1-score—defined as the harmonic mean of precision and sensitivity—for visit durations and event detections (i.e., entries and exits), and the coefficient of determination (r²) for visit counts. Wintergarden showed the highest performance (84 % F1-score, 93 % r²). Metal perch achieved F1-scores of 79 % and 86 % for access and leaving events. Nest boxes showed intermediate performance (78 % F1-score, 77 % r²), while drinkers and feeders were lower (64 % F1-score each; r² values of 69 % and 49 %). These findings confirm RFID’s potential for tracking visits to wintergardens, perches, and nest boxes—demonstrating sufficient performance for practical use, though further optimization through antenna positioning remains possible. For feeders and drinkers, however, accurate tracking remains challenging, and complementary technologies may be required, as rapid movements reduce tag dwell time, overcrowding causes signal interference, and open areas increase misreads from nearby surrounding movement. This study highlights RFID’s value for behavioral research at the individual level in poultry and supports research-driven innovation in housing equipment design. It also demonstrates how a computer-assisted approach can facilitate validation across diverse behavioral contexts.

## Introduction

The interactions of chickens with functional resources—such as drinkers, feeders, perches, nests, and enriched outdoor runs—are crucial for the expression of their natural behaviors and for meeting their physiological needs. These interactions are fundamental for ensuring chickens’ welfare, as they directly impact both their affective states and physical health (Lay et al., 2011). Optimizing access to these resources also benefits farmers by reducing the incidence of health and behavioral problems (e.g., feather pecking), and decreasing associated veterinary costs, thus improving poultry productivity and farm profitability (Europe Food Safety Authority, 2005; Nicol et al., 2013).

However, studying individuals’ resource use remains a significant challenge. Traditional monitoring methods primarily rely on direct human observation and onscreen video analysis. While these approaches are common, they present significant limitations: they are not only time-consuming but also subject to potential errors due to human fatigue (Girard and Cohn, 2016; Alarcón-Nieto et al., 2018), and they are impractical for long-term continuous monitoring in complex environments such as poultry barns. To overcome these limitations, Radio Frequency Identification (RFID) technology offers a promising and effective solution for research applications. RFID provides several advantages over traditional behavioral tracking methods. These include remote monitoring, independence from lighting conditions and line-of-sight, as well as the long lifespan of passive RFID tags, which do not require batteries (Brown-Brandl et al., 2019). Furthermore, RFID has been successfully used to study not only resource visits and health conditions but also social interactions within flocks. For instance, Richards et al. (2012) demonstrated, using RFID tracking, that hens with keel bone fractures had reduced access to outdoor resources, while Gómez et al. (2022) used movement data derived from RFID tracking to model social associations among hens.

The adoption of new animal behavior tracking technologies is crucial for advancing research and practice, but it is essential to ensure that the information they provide is valid and reliable (Martin and Bateson, 2007). Validation is a necessary step in the implementation of any new system; however, it presents several significant methodological challenges.

Traditionally, systems are validated against human-coded observations, a process that is not only time- and labor-consuming—as illustrated by Sales et al. (2015), who logged over 256 h of manual video annotation, describing it as “time-intensive”—but also vulnerable to fatigue and inconsistency (Anderson and Perona, 2014). Achieving reliable coding often requires expert training, repeated calibration, and high inter-rater agreement (Bakeman and Quera, 2011; Hallgren, 2012). Even trained observers may struggle due to the ambiguous or context-dependent nature of behaviors (Altmann, 1974; Martin and Bateson, 2007).

Another major challenge lies in the lack of universally accepted validation frameworks. In the absence of standardized metrics or methodological guidelines, validation reports vary widely in how they assess the performance of animal behavior tracking technologies—some relying on quantitative indices, others on qualitative assessments. The metrics themselves are often calculated differently across studies, which renders cross-study comparisons difficult, if not meaningless (Alindekon et al., 2023).

In response to these challenges, computer-assisted, video-based tools—such as those using spatial tracking or automated annotation—offer a promising alternative by reducing the burden of human coding. Yet, these tools face limitations. Many remain costly (Dell et al., 2014), require technical customization, and continue to struggle with reliable individual identification under markerless conditions—a task complicated by frequent identity switches, complex environments, and large group sizes. While some progress has been made (e.g., Pérez-Escudero et al., 2014; Walter and Couzin, 2021), robust, markerless individual identification in field conditions remains underdeveloped.

Taken together, these challenges may explain why nearly half of RFID-based studies in poultry behavior did not mention independent validation (Alindekon et al., 2023).

The primary goal of this study is to develop and validate an RFID system for tracking chickens in a controlled environment by monitoring their interactions with key functional resources. Here, we present the design and implementation of a novel RFID system, developed based on general guidelines described by Alindekon et al. (2023), followed by a comparison of the collected data with ArUco annotations to evaluate the system’s validity and reliability. ArUco-based annotation, implemented as a computer-assisted video tracking system, was chosen as the validation benchmark. This method is both robust and well-established, and it closely aligns with human observations (95 % consistency), as shown in our previous study (Alindekon et al., 2025). That study was conducted on laying hens using the same mobile barn, the same camera setup, and the same body-worn markers. In addition to its accuracy, the ArUco system enabled annotation that was approximately 5.4 times faster than manual scoring. Crucially, it also allowed for consistent identification of individuals over time, further supporting its use as a practical and efficient validation reference.

## Materials and methods

### Animals, housing and husbandry

#### Animals and ethical considerations

The study was approved by the Animal Experimentation Ethics Committee of Mecklenburg-Pomerania, under the number AZ 7221.3-18196_23. Twenty-one 45-week-old Lohmann Brown laying hens, acquired from a commercial farm, were used.

#### Characteristics of barn and resources

The hens were housed from 4 September 2024 to 30 October 2024 in a mobile poultry barn (ROWAv 200 V 4.0) at the Friedrich Harms Animal Experimentation Station, University of Rostock, in Dummerstorf, Germany. The barn consisted of two compartments: a 16 m² main barn and a 14 m² wintergarden, which provided a semi-open space for scratching and dust bathing. The pophole to the Wintergarden measured 120 cm in width and 35 cm in height, with a 10 cm wide plastic barrier for the chickens to cross. In the main barn, a wooden nest box system was arranged in two rows of six individual nests. Each nest measured 28 × 38 × 25 × 50 cm, with a sloped plastic bottom that allowed eggs to roll forward into a collection area. The bottom of each nest was lined with wood shavings. The same material was used as litter to cover the floor in front of the nest area. A raised plastic slatted area in the main barn also housed various resources. Two linear plastic feed troughs, each 0.5 meters long and accessible from both sides, were used. The drinking system included 2 cups and 12 nipple drinkers along a 3-meter drinker line, positioned 50 cm above the plastic slatted flooring. Two types of perches were available: a 2.25-meter cylindrical metal perch and an L-shaped wooden perch, with two lines.

#### Animal management

The hens were fed 3 to 5 kg feed (PANTO® LMK Legemehlkorn, Germany), split into two portions: one at 7:30 AM and the other at 5:30 PM. The hens were visually inspected twice daily, with a focus on the group's overall behavior (e.g., general activity levels, signs of excessive aggression or abnormal pecking, vocalizations) and appearance (e.g., plumage, signs of injury). The barn was lit for 16 h per day, from 5:30 AM to 9:30 PM. The wintergarden, where the hens had exclusive access to a box containing a mixture of zeolite and fine sand for dustbathing, as well as fresh air, direct sunlight, and ample space for foraging, was automatically opened from 9:30 AM to 8:30 PM. The chickens were acclimated to this environment for six weeks before data collection began.

### RFID development and deployment

The development and deployment steps follow the guidelines of Alindekon et al. (2023), which detail the procedures.

#### Tracking objectives

We intended to develop an RFID system that collects data on the presence of individual chickens within the functional areas of key resources (feeder, drinker, perch, nest boxes, and the wintergarden), referred to as areas of interest (AOIs). The RFID system should record timestamps for entry and exit—and for some resources, continuously register presence on a second-by-second basis—enabling the calculation of frequency, duration, and resource visits over time.

#### Tracking requirements and equipment selection

Selecting RFID components required careful consideration of technical constraints, operational conditions, and compatibility with our tracking objectives. To ensure technical robustness and optimal system integration, we adopted a cross-disciplinary approach, as recommended by Siegford et al. (2023). We collaborated with specialized RFID engineers (Wilmsmeier Solutions, Germany) to ensure full alignment between technical requirements and our research objectives. The details of the selected equipment and the rationale behind each choice are presented below.

• ***Antennas.*** Due to the linear nature of most resources, a linear antenna was required to cover the length of these resources. The antennas needed to be flexible, allowing them to be easily shaped and installed along the linear structures of the barn. Therefore, LOCFIELD® cable-like antennas (FEIG Electronic, Germany) were chosen. These cable-like antennas generate a cylindrical, localized electromagnetic field, adjustable from 1 cm to 2.5 meters, with an active length of at least 2 meters, providing an omnidirectional reading profile. The antenna models were selected based on the length of the resource: ID ANT.U LOCFIELD® Type: 200-250-28-EU used for the interior of the nests, along the underside of the drinker line, and along the popholes leading to the wintergarden; ID ANT.U LOCFIELD® Type: 200-300-50-EU used for the feeders and perch lines.
• ***Readers.*** The system needed to manage simultaneous tag detection when multiple chickens were within the read range, and also to accommodate high-speed reading in areas such as the pophole and nest entry. This required selecting readers capable of handling mass readings and equipped with an anti-collision protocol—a mechanism that ensures signals from different RFID tags do not interfere with each other, allowing each tag to be accurately identified even in high-crowding zones. Additionally, the system required a reading range extending from close contact to a distance corresponding to the body length of a chicken, estimated at around 30 cm. To meet these requirements, the FEIG LRU1002 Fixed Long Range UHF RFID Reader (FEIG Electronic, Germany), operating in the 865–868 MHz range as required by European ETSI (European Telecommunications Standards Institute) regulations, was selected.
• ***Tags.*** Given the challenging environment, including the presence of manure and dust, and compatibility with the selected reader, the tracking conditions required finding tags designed to operate in the UHF range. These tags needed to be made of materials that provide elasticity and resistance to poultry manure and pecking, as well as be lightweight to avoid compromising the chickens' well-being. The FLEXTAG 62 × 12-TPU-YE (HellermannTyton, Germany), made of thermoplastic polyurethane and weighing only 3 g, was selected to meet the needs of our study.
• **Coaxial Cables.** The criteria were to ensure a uniform cable type and length to guarantee consistent signal transmission and power settings across the system, minimizing signal loss. All antennas were connected to the readers using ID ISC.ANT.C6-A UHF-Antennenkabel SMA/SMA 6 m coaxial cables (FEIG Electronic, Germany).

#### Tag ‘N track RFID software solution for data management

As with equipment selection, developing RFID data management software involves challenges that require technical competencies not typically found in animal behavior specialists. To address these challenges, we partnered with software engineers (Wille Engineering, Germany) to create a custom solution called Tag ‘N Track. This program, built using an API and integrated with middleware from the RFID reader manufacturer, tackles key technical issues such as time synchronization across all readers, data buffering, and the implementation of a core algorithm for detecting enter/exit events (with each RFID tag generating an event every 1000 ms).

From the outset, the software was designed to use the system clock on the host computer for timestamping. This allows the RFID system to be integrated with, and synchronised to, any other tracking system installed in parallel (e.g., a camera system for validation purposes).

The software provides real-time data visualization, efficient storage via InfluxDB’s UI Data Explorer, hardware monitoring to prevent data loss, and up-time monitoring with alerts. Supplementary Material A provides an overview of the query interface in InfluxDB’s UI Data Explorer, with a screenshot showing RFID event data visualization as an example.

#### System architecture

**Fig. 1:**
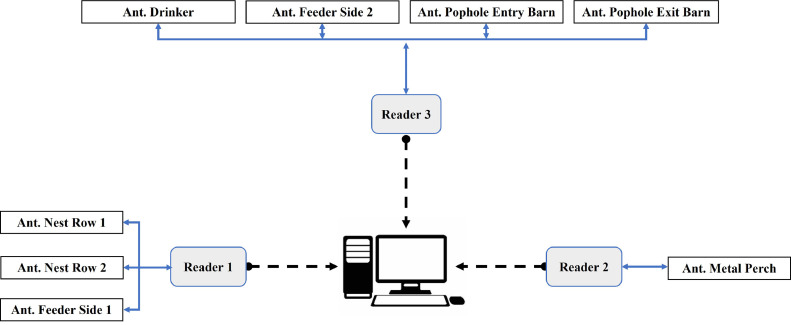
System architecture of the RFID system to monitor individual chickens' presence within functional areas of key resources. The blue line with two-headed arrows represents the coaxial cables connecting the reader and the antennas (Ant.). The dashed arrows correspond to the ethernet cable connecting the reader and the host computer.

The goal was to have an architecture that allowed for efficient and synchronized data collection across all functional areas in the barn. In total, three readers were used. Each reader operated as a standalone subsystem and was connected to a certain number of antennas linked to resources of focus. The data from the various readers were then centralized through a network to a hosting computer for storage and analysis (Fig. 1).

#### Tag attachment to chickens

**Fig. 2:**
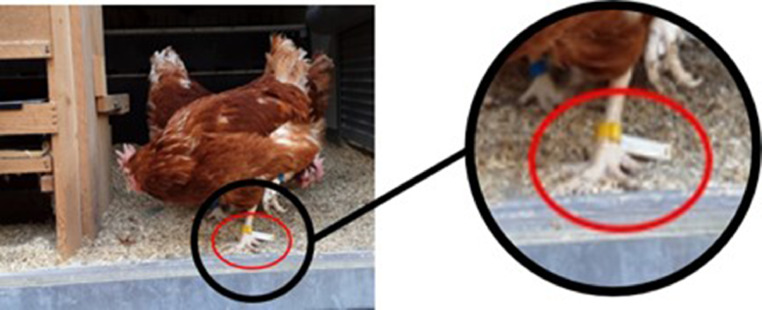
An RFID tag is attached to a standard chicken leg ring and fixed to the ankle in a flag-like position, perpendicular to the tarsus. The RFID tag and attachment point are marked with a red circle.

The RFID tags were glued to a standard chicken identification ring and fitted to the chickens' ankles in a flag-like position. Positioning the tags in this way was intended to maximize the contact surface with the cylindrical electromagnetic field generated by the antennas and help minimize interference from body tissue (Fig. 2).

#### Antenna placement on focal resources

**Fig. 3:**
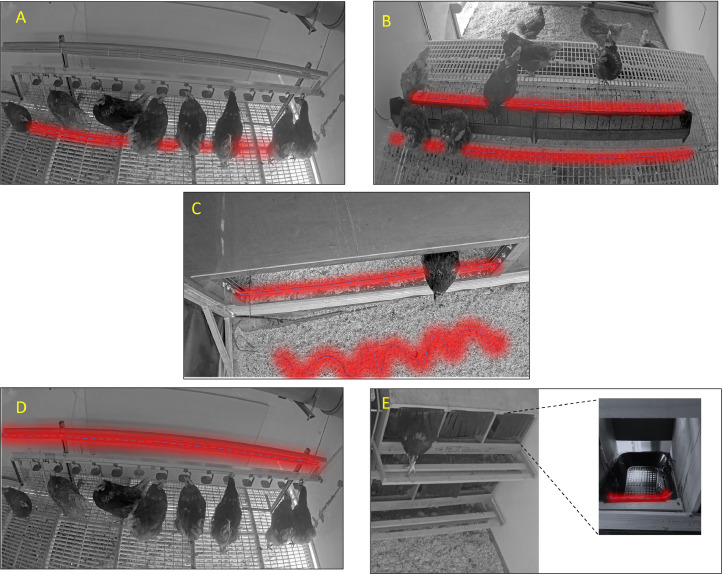
Placement of antennas and expected signal distribution (marked in red) along them to register chickens in the resources' functional areas. The figure shows in A: Antenna covering the drinking line; B: Two antennas in the feeding area; C: Two antennas for registering Barn-to-Wintergarden and vice versa; D: Antenna for the metal perch line; E: Antenna deployed across nest boxes.

The antennas were deployed to optimize the electromagnetic field coverage, ensure reliable readings, and minimize the risk of missed RFID tag readings in the functional areas of the resources where the chickens position themselves for use (Fig. 3).

• **Metal Perch.** An antenna was installed beneath the metal perch line at a distance of 7.5 cm from the metal. It was fixed on a wooden slat attached below the perch and enclosed in a plastic tube to prevent direct contact with both the metal and chicken droppings, thereby reducing potential signal interference.
• **Feeding Area.** Two antennas were placed along the feed troughs, fixed directly to the slatted floor on both sides, at the chickens' foot level, ensuring independent coverage on each side, regardless of which side the chickens approach.
• **Pophole Passage (Barn to Wintergarden and vice versa).** Two antennas were installed to register the passages between the barn and the wintergarden. The first antenna is placed on the upper edge of the barrier that the chickens must cross, while the second antenna is positioned on the other side of the wintergarden, arranged in a serpentine antenna layout, in order to extend the electromagnetic field coverage and ensure more effective capture of the chickens' passage.
• **Drinking Area.** An antenna was installed beneath the drinker line on the slatted floor, protected by PVC to shield it from moisture and water runoff.
• **Nest boxes.** A single RFID antenna was placed at the interior entrance of the nest, positioned near the bottom and extending through each row to detect entry and exit timestamps as chickens moved in and out. The antenna was arranged in a sinusoidal pattern, vertically aligned on a single level, to ensure coverage of all individual nest boxes.

#### Operating parameters of the readers

The power settings applied were informed by preliminary installation and simulation tests conducted without animals, using the FEIG ISOStart+ software (Version 11.05.00), a configuration and testing tool provided by FEIG Electronic for their RFID readers. The tests involved 10 tags placed simultaneously at 10 cm intervals along a linear axis, repeated twice over five iterations.

For feeders, the drinker, and nest boxes, RFID readers were configured at 0.1 W, which is also their minimum allowable power setting. At this level, tag detection rate was consistently achieved at 100 % up to approximately 10 cm, began to decline around 15 cm, and dropped to zero beyond 30 cm. This range was considered sufficient for the intended applications, and 0.1 W was therefore maintained in these areas.

In contrast, for specific locations such as the metal perch and the pophole leading to the wintergarden, the power level was increased to 0.2 W in order to achieve 100 % tag detection. This adjustment was made to account for potential signal interference caused by metal structures (in the case of the perch), the positioning of the readers, and the need to capture rapid animal movement. The physical layout of these zones—such as elevated perches and pophole crossing points—helped minimize the risk of unintended tag detection, even at the slightly higher power level.

The readers operated at a fixed read rate of one read per second, and the EPC Gen 2 anti-collision protocol in dense mode was enabled to ensure reliable identification in shared-use areas where multiple animals could be present simultaneously.

#### Processing raw data of RFID annotations

Since the nature of raw RFID data varied across resources, the processing approach was adapted accordingly to ensure accurate identification and quantification of visit events.

• **Feeder and Drinker.** The raw data from the RFID data management system for these resources were highly fragmented, with visit sequences characterized by numerous short and isolated events, separated by momentary interruptions. To achieve a more reliable and consistent quantification of visits, we applied a bout-merging criterion for each resource—a temporal threshold used to link consecutive RFID detections into a single visit event. These thresholds were defined based on the average presence durations in the feeder and drinker areas, as measured using the gold standard video system.
  After applying the bout-merging criteria, the first timestamp within each merged detection sequence was assigned as the entry time, and the last timestamp as the exit time for that visit. This approach allowed us to extract coherent visit events from otherwise fragmented RFID readings.
After applying the bout-merging criteria, the first timestamp within each merged detection sequence was assigned as the entry time, and the last timestamp as the exit time for that visit. This approach allowed us to extract coherent visit events from otherwise fragmented RFID readings.
• **Nest box.** Raw data from RFID antennas placed at the interior entrance of the nest boxes were used to determine visit events. For each individual, the first detection within the morning time window was considered the entry timestamp, and the last detection before exiting the nest was used as the exit timestamp. The visit duration was calculated as the time difference between these two points. Since the antennas were located inside the nest, intermittent signal loss during occupancy was expected and intermediate detections were disregarded.
• **Wintergarden.** We adopted an indirect approach based on the sequential order of RFID detections to identify visit events and calculate their count and duration. First, we distinguished between the RFID antennas covering the barn interior and the single RFID antenna positioned inside the Wintergarden, right at its entrance. To define a complete visit, we applied a double-transition logic: when a chicken was first detected by an antenna inside the barn and then at the Wintergarden RFID point, this indicated a "Barn to Wintergarden" transition. Conversely, if the first detection occurred in the Wintergarden and was followed by one in the barn, this signified a "Wintergarden to Barn" transition. Only sequences including both transitions were used to determine visit metrics. By calculating the time difference between each Wintergarden entry timestamp and the subsequent exit transition, we determined both the total number of visits and the duration of each visit.
• **Metal Perch.** To determine nighttime perch occupancy, we first identified the time of access (i.e., entry) using the timestamp of the first RFID detection on the perch after the evening feeding period (served at 5:30 PM). This moment typically corresponds to hens jumping onto the perch while still in a standing position, which would be expected to allow more reliable detection. Once perched in a sitting position, however, RFID tracking becomes less effective: the hens’ body posture and weight press the leg-mounted tag against the perch and may block the antenna’s magnetic field, leading to signal loss or gaps in detection. The time of perch leaving (i.e., exit) was defined as the last RFID detection recorded before the next morning feeding period. Since hens usually stand up before leaving the perch, this posture would be expected to permit tag detection again. This method assumes that hens remain perched through the night, making the first detection after evening feeding and the final detection before morning feeding useful indicators of nighttime perch access and leaving.

### Benchmarking video recordings and 3D-ArUco marker annotations

To facilitate the validation of the RFID system during deployment, a computer-assisted, video-based method involving 3D-ArUco Marker Annotations was used. The technical details regarding the cameras used, installation procedures, and 3D-ArUco marker tracking are provided in our previously published paper (Alindekon et al., 2025), where we also detail the high reliability of this benchmarking system, which is used here as the gold standard. The practical recommendations outlined in that paper have also been applied in the present study.

#### Camera installation and video recording

The cameras were positioned to focus on each key resource used by the chickens, and continuous video recording was conducted throughout the entire lighted period of the day (5:30 AM to 9:30 PM) from 15 October 2024 to 19 October 2024. For the analysis, however, we extracted specific video segments corresponding to fixed time windows, applied consistently across days for each resource.

The selection of these time windows—considered as peak activity periods—was guided by preliminary observations during pilot recordings, daily management and intervention routines within the barn, and supported by findings from the poultry behavior literature, notably the review by Appleby et al. (2004). This approach allowed us to focus on periods of high activity and reduce the inclusion of low-interest events (negatives).

The selected hours for each resource were as follows:

• Drinker: 8:00 AM – 10:00 AM and 2:00 PM – 4:00 PM
• Feeder: 6:00 AM – 7:00 AM, 8:00 AM – 10:00 AM, and 6:00 PM – 7:00 PM
• Nest: 6:30 AM – 10:30 AM
• Metal Perch: 5:30 AM – 8:30 AM and 7:00 PM – 9:30 PM
• Pophole: 9:00 AM – 1:00 PM

In total, 16 h of video recordings per resource were obtained (four hours each day), except for the metal perch, which, due to the lighting schedule, was recorded for 2.5 h for the evening entry and 3 h for the morning exit events.

Video timestamps were generated using the system clock of the host computer, which also handled RFID data collection, ensuring a shared time base between both systems, so that any discrepancy would reflect RFID reading performance rather than a time misalignment.

#### Definition of areas of interest (AOIs) around resources for video annotations

The AOIs referred to functional areas defined in the videos, representing the spaces where the chickens actively engaged with the resources (i.e., effectively used them). This process involved marking the resource users from multiple selected frames, outlining the functional areas, and blurring non-relevant areas. The resource-specific overlay was then applied to the corresponding videos, ensuring that only the hens within the designated areas of interest were visible for presence tracking. Details on the procedure for defining AOIs are provided in Alindekon et al. (2024; 2025).

#### 3D ArUco marker tracking and visit event identification

During the video recording, the chickens wore vests equipped with 3D ArUco markers, similar to QR codes, each associated with a unique numeric identifier. These markers were automatically detected in each frame of the videos using OpenCV version 4.10.0, run in Python (version: 3.11.4). With the frame rate of each video known, it was possible to calculate the duration of presence for each unique ID in the designated areas of interest (AOIs). This allowed the automated collection of timestamps for entries and exits, as well as the duration of presence within the AOIs, which was saved in a CSV file. To infer resource visits based on raw data from ArUco detection within the AOIs, specific considerations were made according to the nature of the resources:

• **Feeder and Drinker**. For these resources, the visit duration of each individual was determined based on their presence within the AOI assigned to each resource.
• **Wintergarden**. Access to this resource was through a pophole, with AOIs defined on the barn side and on the Wintergarden side. The direction of passage was inferred from the sequence of detections: if the first detection occurred on the barn side and the next on the Wintergarden side, movement was considered from barn to Wintergarden. Conversely, if detection began on the Wintergarden side and was followed by one on the barn side, the movement was considered from Wintergarden to barn. Using this directional information, and applying the same double-transition logic as in the RFID data, we inferred visits as sequences involving both an entry and a subsequent exit**.** This allowed us to calculate the number and duration of visits to the Wintergarden.
• **Nest**. Due to space constraints and the wide camera angle, our setup was limited to monitoring the nest entrances rather than the interiors of the nest boxes. We inferred visits indirectly based on the presence of chickens at the nest entrance. Since the 3D ArUco-based system only captured presence on the nest access ramp without specifying the direction of movement, it was unclear whether a chicken disappearing from view had entered the nest or jumped onto the floor. Therefore, minimal human intervention was used to verify all exit events flagged by the ArUco-based system, ensuring that only confirmed nest entries were retained. Verification consisted of manually checking 128 flagged exit events; of these, 79 were confirmed as true nest entries and 49 were excluded as birds descending from the ramp. This verification step was quick (completed in a few minutes), objective, and required no behavioral interpretation—only the use of timestamps to locate the flagged event in the video and confirm the direction of movement.
• **Metal Perch.** Tracking perching over time was not feasible due to suboptimal camera installation. The camera’s view was limited to a side angle, and a top-down perspective of the perching animals was not possible. Due to the sloped roof of the barn and the height of the perch, it was challenging to capture the full body of an animal once it was perched. When the chickens settled for the night, their feathers obscured the front part of the marker, further complicating continuous tracking. Moreover, the absence of light during the night made the video recordings unusable for monitoring night perching. As a result, tracking on the perch was limited to detecting timestamps for access in the evening and exit in the morning, with no continuous tracking of presence or duration during perching.

**Fig. 4:**
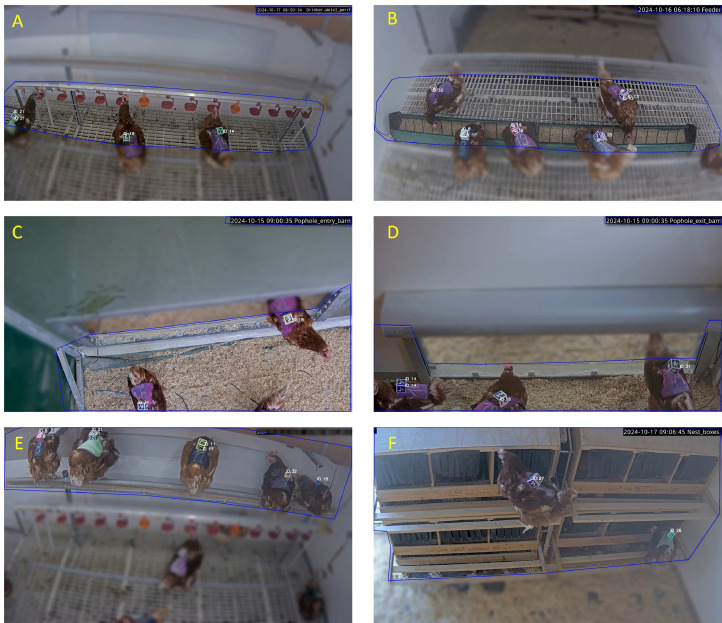
The area of interest (AOI, non-blurred zone) for 3D-ArUco annotations used as the ground truth for validating RFID annotations. Markers on the chickens present are automatically identified, and their IDs are displayed from the moment they enter and throughout their presence in the AOI. The figure shows in A: AOI defined for the drinking line; B: Two AOIs in the feeding area; C and D: AOIs covering Barn-to-Wintergarden and vice versa, respectively; E: AOI defined for the metal perch line; F: AOI defined for nest boxes.

Fig. 4 presents video snapshots showing defined AOIs, representing the functional areas for each resource, along with the marker IDs displayed after the automated detection of presence at each resource. The ArUco-based tracking tool used for automated detection of presence served as a benchmark for validating the RFID outputs.

### Alignment of processed RFID and ArUco-based data and metrics calculation

Since the nature of data varies across resources (entry and exit timestamps for the perch and visit durations for other resources), different approaches for alignment and metrics calculation were applied based on each specific case.

#### Duration-based metric calculations

This approach was applied to the feeder, drinker, nest box, and wintergarden. Alignment between RFID and ArUco data was performed on a second-by-second basis by matching timestamps, allowing the computation of confusion matrix elements for each individual and each resource at every time point. These elements included True Positives (TP), False Negatives (FN), False Positives (FP), and True Negatives (TN).

From these, standard performance metrics can be derived. However, we selectively focused on those most relevant for evaluating detection quality. We computed Precision (TP / [TP + FP]), Sensitivity (TP / [TP + FN]), and their harmonic mean, the F1-score (2 × [Precision × Sensitivity] / [Precision + Sensitivity]). The F1-score was chosen because it combines both precision and sensitivity into a single value, providing a balanced measure of the system’s ability to correctly detect actual events while minimizing both missed detections and false positives. Its calculation excludes true negatives and therefore offers a more meaningful evaluation of detection performance in the context of animal behavior.

Accuracy, by contrast, reflects the overall proportion of correct decisions—whether detecting a presence or correctly identifying an absence. Unlike the F1-score, it includes true negatives in its calculation and was therefore not used in our analysis. In behavioral monitoring, where animals often spend long periods away from a focal resource, true negatives can dominate the data. This can lead to misleadingly high accuracy values, even when the detection of actual events is poor (Adrion et al., 2020; Alindekon et al., 2023). For this reason, accuracy was deemed inappropriate for our validation goals.

#### Visit count-based metrics calculation

This approach was also applied to the feeder, drinker, nest box, and wintergarden. Here, the total number of visits per individual were compared between the two methods to quantify how well they agreed for each resource. This involved identifying the correlation between RFID-based and ArUco-based outputs and then computing the coefficient of determination, which was used as an indicator of agreement and reliability.

#### Timestamps difference-based metric calculation

This approach was applied exclusively to the Metal Perch. Based on the validation method described by Gebhardt-Henrich et al. (2023), we adopted a 60-second matching window between RFID and gold standard timestamps. If the time difference between the two timestamps was ≤ 60 s, the event was considered a match. Events were classified as follows: TP (True Positive, when the time difference met the threshold), FP (False Positive, when RFID detections had no corresponding ArUco match), and FN (False Negative, when ArUco detections had no corresponding RFID match). Finally, Precision, Sensitivity, and F1-score were computed separately for both entry and exit events.

## Results

### Overview of collected data

Since the data-collection approach differed by resource, we broadly grouped the resources into three categories.

#### Feeder and drinker

**Fig. 5:**
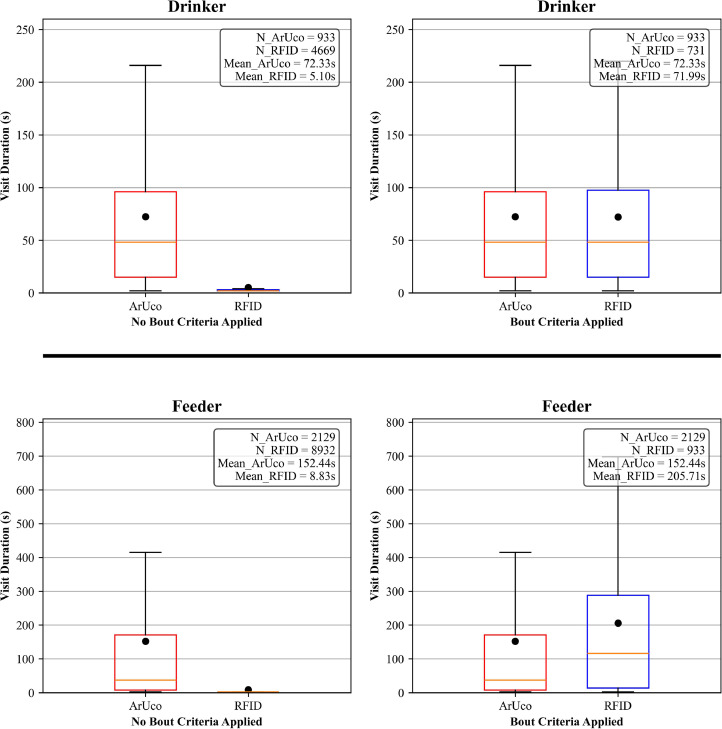
Effectiveness of bout criteria application on RFID visit data. The average presence duration according to the ArUco-based annotation (73 seconds for the feeder, 153 s for the drinker) was used to reduce fragmented, short-duration visits interspersed with RFID gaps. N_Aruco and N_RFID refer to the number of visits recorded by each approach, while mean_Aruco and mean_RFID represent their respective average durations (in seconds).

Without bout merging, the RFID-based visits for the feeder and drinker were highly fragmented, resulting in numerous short-duration visits—unlike the ArUco-based annotations, which produced fewer, more continuous visits. However, when we applied bout-merging criteria to the RFID data (153 s for the feeder and 73 s for the drinker, derived from ArUco detections), these previously fragmented RFID visits were consolidated, yielding visit counts and durations comparable to those recorded by ArUco. Specifically, bout merging increased the mean visit duration for the drinker from 5.10 s to 71.99 s, closely matching the ArUco-based average of 72.33 s. For the feeder, the mean duration rose from 8.83 s to 205.71 s, approaching the ArUco-estimated average of 152.44 s (Fig. 5). These adjustments also significantly reduced the number of RFID-detected visits, enhancing consistency between the two systems. RFID data, incorporating these bout criteria, were used for both resources throughout the rest of the study.

#### Nest boxes and wintergarden

For these resources, the RFID- and ArUco-based visit counts were similar, indicating no need for additional bout-merging criteria. Table 1 summarizes the results for these two resources.

**Table 1:** Overview of Nest box and Wintergarden visits. The data presented are the mean duration per visit in seconds (mean ± SD) and the total number of visits recorded by both RFID and ArUco across all individuals and days of data collection. No bout-merging criteria were applied.

**Resources**	**Mean duration (s)**		**Total Visits**	
	**ArUco**	**RFID**	**ArUco**	**RFID**
Wintergarden	2227.65 ± 2230.88	1637.90 ± 1888.05	176	180
Nest boxes	1900.92 ± 960.73	1789.94 ± 1638.55	77	79

#### Metal perch (access and leaving)

For the metal perch, the differences between the RFID and ArUco timestamps at the occurrence of each event were 125.32 ± 410.40 s for entry events and 25.61 ± 37.18 s for exit events.

The supplementary material B provides a visual comparison of RFID- and ArUco-based resource visit events, using a single chicken as a representative example.

### Performance metrics

#### Performance metrics based on visit durations or occurrence timestamps

Based on the F1-score, which balances precision and sensitivity, the performance metrics revealed notable differences among the resources (Table 2). Wintergarden usage exhibited the highest performance with an F1-score of 0.84. For the metal perch, the system achieved high F1-scores: 0.79 for perch access and 0.86 for morning perch leaving, with the matching window between RFID and ArUco timestamps set to 60 s. Nest boxes followed with an F1-score of 0.78. Lastly, both the drinker and feeder displayed relatively lowest F1-scores of 0.64 (Table 1), with high precision values (0.86 and 0.73, respectively) but low sensitivity (0.51 for the drinker and 0.57 for the feeder).

**Table 2:** Performance metrics (Precision, Sensitivity, and F1-Score) of RFID-based tracking for different key resources, benchmarked against 3D-ArUco annotations.

**Metrics**	**Wintergarden**	**Perch leaving**	**Perch access**	**Nest boxes**	**Drinker**	**Feeder**
Precision	0.98	0.85	0.82	0.80	0.86	0.73
Sensitivity	0.74	0.87	0.75	0.77	0.51	0.57
**F1-score**	**0.84**	**0.86**	**0.79**	**0.78**	**0.64**	**0.64**

#### Visit count-based performance metrics

**Fig. 6:**
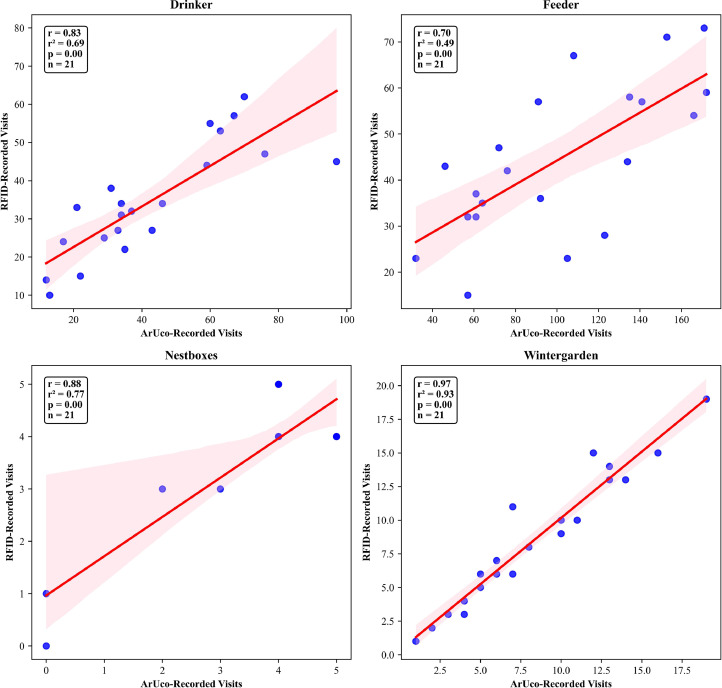
Correlation between total ArUco and RFID visit counts per chicken over the entire data collection period. “r” represents Pearson's correlation coefficient and “r-squared” is the coefficient of determination.

All resources exhibited correlations between ArUco and RFID visit counts above 0.70, but across resources, the correlations varied, with the strongest agreement observed in Wintergarden (Fig. 6). The coefficients of determination indicated that, for most of these resources, a substantial portion of the variance in RFID visits was explained by video visits. In Wintergarden, 93 % of the variance in RFID visits was explained by video visits. In Nest boxes and Drinker, 77 % and 69 % of the variance were explained, respectively. Feeder exhibited the weakest alignment between the two recording methods, with video visits accounting for only 49 % of the variance in RFID visits.

## Discussion

This study investigated the reliability and validity of an RFID system in capturing the interactions of chickens housed in a mobile barn with several key functional resources, such as drinker, feeder, perch, nest boxes, and wintergarden. The RFID system was benchmarked against a computer-assisted, automated 3D ArUco-based video annotation system. Our findings revealed significant differences in RFID performance among the resources. Overall, the monitoring of visits with RFID to the wintergarden, metal perch (within the defined temporal threshold), and nest boxes can be considered acceptable, as these provide a reliable balance between precision and sensitivity. However, for drinkers and feeders, RFID requires adjustments to improve accuracy.

### Transitions and visits in wintergarden monitoring with RFID

Using the proposed setup, the RFID accurately identified 9 out of 10 wintergarden visits (93 % coefficient of determination). In terms of visit duration, for the 100 s indicated by the gold standard as an effective visit, the RFID approach successfully captured 74 of those seconds (sensitivity = 74 %), while 98 % of the RFID-detected seconds were correct (precision = 98 %). These results confirm the efficacy of the RFID system for monitoring wintergarden visits and align with findings in the literature. For instance, Thurner et al. (2010) reported a 98 % identification rate for outdoor range usage when comparing RFID with video recordings of pophole passages, while Gebhardt-Henrich et al. (2014) achieved 94 % accuracy in detecting entries relative to exits.

The successful monitoring of wintergarden visits using only RFID can be attributed to two main factors. First, the integration of multiple RFID antennas provides redundancy, which, as demonstrated in Gebhardt-Henrich et al. (2023), significantly improves sensitivity. In their study, deploying multiple antennas in transition zones resulted in RFID data matching the observational data at a rate of 93 %, compared to only 39 % for an isolated antenna. Second, implementing a double-transition principle—where a passage is validated only if identification occurs sequentially at an entry and an exit—minimizes false readings (Siegford et al., 2016).

RFID-based monitoring of wintergarden visits offers a valuable tool for optimizing management practices and enhancing poultry welfare. First, RFID facilitates the development and evaluation of enrichment strategies in the wintergarden by assessing individual usage frequency and enrichment preferences—such as monitoring the provision of insects as supplementary feed (Ruhnke et al., 2018). Next, it identifies key factors—such as the season (e.g., winter or summer) and the time of day (Taylor et al., 2017)—that significantly influence outdoor use, thereby enabling managers to fine-tune resource access. Finally, RFID-based monitoring provides essential behavioral insights for fundamental poultry studies by revealing both intra- and inter-individual behavior patterns (Larsen et al., 2017; Campbell et al., 2018) and elucidating how chickens form social associations under commercial farming conditions (Gómez et al., 2022).

### RFID for monitoring nighttime perch access and leaving

Using a 60-second matching window between RFID and ArUco timestamps, the RFID system showed satisfactory performance in detecting metal perch use during the night. Out of 100 events indicated by the gold standard, the system successfully identified 75 perch access events and 87 leaving events. Exit events were detected more reliably than entries, as reflected by the much lower standard deviation in timestamp differences (37.18 s vs. 410.40 s). A plausible explanation is that birds tend to exhibit more variable and unstable movements when accessing the perch—such as shifting posture, bending their legs, or adjusting balance with the body leaning close to the perch—making initial detection more prone to error. In contrast, leaving the perch generally involves a clearer and more direct movement, which likely contributes to more consistent detection. While the exact reason for this difference in detection sensitivity is unclear, further investigation would be beneficial. Nevertheless, sensitivities of 75 % and 87 %, respectively, can already be considered satisfactory for both event types.

These results are comparable to those reported by Wang et al. (2019), who achieved higher accuracies (97.77 % and 99.88 %) using two RFID tags per bird—an approach that may partly explain their superior performance. Our findings are also in line with Gebhardt-Henrich et al. (2023), who reported 93 % sensitivity in detecting tier transitions. Together, these metrics indicate that RFID can reliably monitor nighttime perching behavior in chickens under certain conditions.

The ability to track nighttime perching behavior reliably with the RFID setup used here stems from two key factors. First, the elevated perch position minimizes false readings by isolating the perch from other resources and interference. Second, precise timestamps capture only brief standing moments—just before settling onto the perch in the evening and immediately before leaving it in the morning—which helps avoid errors associated with sitting postures. Continuous, second-by-second RFID detection would likely be less effective in this context due to intermittent signal obstruction caused by the hen's body tissue (Brown-Brandl et al., 2019). The inability to clearly distinguish actual perch movements from signal interruptions makes the current RFID setup unsuitable for continuous second-by-second monitoring of daytime perch use.

Nonetheless, a second-by-second approach could potentially be achieved through other means. For instance, integrating RFID with complementary technologies such as load cells installed under the perch (Wang et al., 2019) may enable continuous and precise occupancy tracking, even during signal loss. Similarly, low-frequency RFID systems could theoretically support second-by-second detection if technical limitations related to multi-animal detection are addressed. In parallel, alternative tagging strategies—such as attaching tags to body areas that would be less affected by signal blockage in the context of nighttime perching posture (e.g., the neck or wings; Toth et al., 2013; Li et al., 2017)—could also help mitigate signal loss, though such approaches require further validation.

Even in such cases, we would not expect significantly different reliability outcomes between a second-by-second and an event-based approach—at least for nighttime perching, which is the specific context here. Unlike other resources such as feeders or drinkers, where birds interact briefly and irregularly, nighttime perch occupancy is prolonged and stable. Therefore, the nature of the behavior itself supports the validity of using only the first and last detection points as reliable indicators of presence.

Precisely monitoring perch access and leaving timestamps with RFID technology offers numerous research opportunities in ethology and poultry welfare. Social hierarchy significantly influences perch access, with dominant birds often monopolizing elevated perching spaces and displacing subordinate individuals (Cordiner and Savory, 2001; Wang et al., 2022). Since RFID technology accurately identifies the order and timing of successful and unsuccessful perch-use events, it could provide deeper insights into social hierarchy compared to traditional visual observation methods.

Additionally, RFID technology could further support investigations into the links between individual health, welfare conditions, and perch utilization. Precise monitoring can identify individuals that consistently hesitate or take longer to perch or descend, signaling underlying welfare issues such as social stress (e.g., intimidation or exclusion) or health issues (e.g., open wounds, keel bone fractures, foot pad dermatitis).

### Nest box visit monitoring with RFID

The proposed RFID system proved to be generally reliable for monitoring nest box usage, although some errors were observed in recording both usage duration and visit counts. Our results indicate that over three-quarters of the nest box usage durations and visit counts were accurately recorded (F1-Score: 78 % and 77 %).

Compared to the ground truth, our RFID system showed discrepancies in detecting nest visits, as it could not distinguish brief nest inspections from prolonged visits indicative of actual nest use. As long as the chicken remained within the same nest row, the system merged successive detections into a single extended visit, even when the bird briefly exited between detections. In contrast, the video validation could differentiate short inspections from true visits based on movement between rows and detection by another antenna. This limitation stems from the use of a single antenna per row, which prevents separate detection of entry and exit events.

Compared to the literature, our findings are similar to those reported by Sibanda et al. (2020), who documented 80 % sensitivity for detecting chickens in nest box areas, with a coefficient of determination ranging between 53 % and 66 %. However, our performance remains lower than that of Li et al. (2017), who achieved a precision of 91.4 % in bird detection (calculated as the percentage of chickens detected by the RFID system relative to those observed in video images) and a coefficient of determination of 98 %. This discrepancy is likely due to differences in setup. In their study, the authors positioned their antennas beneath the nests and equipped each chicken with two tags—one on the neck and another on the leg. This arrangement likely contributed to their superior performance. In contrast, our study used a single tag attached to the ankle and a single antenna arranged at the interior entrance of the nest.

To improve nest box monitoring, it may be beneficial to increase the number of RFID antennas and strategically place them around the nest boxes. For example, installing an antenna under the access ramp, one at the entrance (as currently implemented), and another beneath the nest box—as demonstrated by Thurner et al. (2008); Zaninelli et al. (2015) and Li et al. (2017)—could significantly enhance system performance.

Additionally, monitoring can be further optimized by integrating weight measurement technology to track individual oviposition events based on body weight fluctuations. Heinrich et al. (2013) successfully recorded oviposition events by combining an RFID system with a weighing system, allowing for precise individual identification and automated oviposition detection.

In addition to hardware improvements, machine learning trained on video-validated data could improve the classification of nest visits. Hens often perform brief, repeated inspections before selecting a nest, particularly during the pre-oviposition period, and these short visits differ in duration and pattern from actual laying events (Cooper and Appleby, 1996). A trained model could use these features to distinguish between exploratory behavior and effective nest occupancy for oviposition. This would improve biological accuracy and reduce misclassification caused by hesitation or nest exploration.

### RFID for feeder and drinker visit monitoring

RFID performance remained suboptimal when tracking both the feeder and the drinker. Considering the drinker, if we look at 100 s identified by the gold standard as an effective visit, the RFID system successfully captured 51 s (sensitivity = 51 %), while 86 % of the RFID-detected seconds were correct (precision = 86 %). For the feeder, out of 100 gold-standard seconds, 57 s were correctly captured (sensitivity = 57 %), and 73 % of the RFID-detected seconds were correct (precision = 73 %). Regarding visit counts, one-third of drinker visits and half of feeder visits went undetected (correlation coefficients: 69 % for drinkers, 49 % for feeders), despite efforts to merge closely spaced registrations and reduce interference from physical objects, metals, liquids, and electrical noise—factors known to degrade UHF RFID signals (Brown-Brandl et al., 2019).

Three main factors contribute to RFID misreads in these areas. First, feeding and drinking behaviors involve short-duration visits (Shynkaruk et al., 2019) and rapid, high-intensity, intermittent movements (Li et al., 2020; Yang et al., 2020), accompanied by frequent positional shifts—leading to detection gaps (Alindekon et al., 2023, 2024). This issue is further compounded by the open and freely accessible layout of these areas, which allows birds to pass through casually without necessarily engaging with the resource—a similar observation was made in pigs by Maselyne et al. (2016). Second, partial positioning of birds can affect signal capture, as individuals may not remain fully within the RFID read range. Third, signal interference from overlapping RFID tags—caused by multiple birds in close proximity—can further hinder consistent detection, particularly in high-crowding zones such as around feeders and drinkers (Alindekon et al., 2023).

Several solutions can be considered to address the limitations of RFID-based detection. One practical approach involves monitoring water and feed intake using individual stations equipped with RFID. As demonstrated by Maselyne et al. (2016) in pigs, this method employs physical partitions to guide animals into well-defined detection zones, reducing ambiguity caused by unstructured access. However, modifying the environment to physically constrain animal movement may disrupt natural behavioral patterns, reducing the practical relevance of RFID-generated data and limiting its applicability in commercial poultry settings.

Another promising strategy is the integration of RFID with vision-based tracking technologies. Although this fusion was not pilot-tested in the present study, it represents a promising direction for future work. For instance, combining YOLO object detection with multi-object tracking algorithms—such as SORT, DeepSORT, or BoT-SORT—can provide continuous and robust animal identification through visual means. These systems have already shown strong performance in animal monitoring contexts (Siriani et al., 2023; Yang et al., 2024; Triyanto et al., 2024). If combined with RFID, they can offer a complementary data stream that may improve the overall accuracy and reliability of behavior tracking. Additionally, visual marker tracking using technologies such as ArUco markers, AprilTags, or QR codes—as applied by Alarcón-Nieto et al. (2018), van Putten et al. (2025), and Alindekon et al. (2025)—can further support individual identification, especially in cases where RFID signal detection is unreliable or incomplete. Future research should validate the feasibility of combining these technologies with RFID to develop an optimized system suitable for commercial poultry farming.

### General limitations of the study

Several constraints related to RFID tracking, reproducibility, and the ArUco-based reference method should be considered as general limitations of this study.

#### RFID-related limitations

One of the primary limitations of RFID tracking is that it detects presence near a resource but does not confirm actual engagement. This is particularly relevant for feeders, drinkers, and nest boxes, where mere presence does not necessarily indicate usage (Doornweerd et al., 2023). For instance, in nest boxes, RFID cannot distinguish between oviposition and non-oviposition visits, such as when a hen merely enters and exits without laying an egg. Similarly, for feeders and drinkers, some RFID detections may reflect hens merely passing by or standing near the resource without actual consumption. Based on our previous study (Alindekon et al., 2024), the proportion of RFID-detected visits that correspond to confirmed usage was estimated at approximately 60 % for feeders and 50 % for drinkers.

Practical considerations for commercial applications must be taken into account. While RFID technology is promising for behavioral monitoring, its implementation is currently more feasible in controlled research environments than in large-scale commercial settings. Several factors influence RFID efficiency in real-world applications. In this study, the system was deployed in a specific experimental barn, where customized materials (e.g., wooden nest boxes instead of metallic ones) were used to minimize interference and ensure accurate RFID detection. Furthermore, the small flock size and low animal density provided better tracking conditions. In contrast, commercial farms typically have higher stocking densities and limited space per animal (Leone and Estévez, 2008), which could significantly impact RFID tracking performance.

#### Methodological considerations

Beyond RFID system constraints, certain methodological aspects of the study should also be acknowledged. One key factor is the variability in the criteria definition and performance metric calculations across studies. The bout criterion varies significantly in the literature (Thurner et al., 2008; Li et al., 2017, 2019; Wang et al., 2019), affecting comparability. Some studies test multiple thresholds and select the one that maximizes performance metrics, while others rely on fixed values based on prior research or an error minimization approach. In this study, we applied a bout threshold derived from the average visit duration obtained from ArUco annotations. Similarly, the calculation of performance metrics (e.g., precision, sensitivity, and F1-score) differs across studies, further complicating comparisons (Alindekon et al., 2023). Future research should carefully consider the bout criteria selection and performance metric calculations to enhance reproducibility and comparability with our findings.

Another methodological limitation arises from the ArUco-based validation approach, which has two major constraints. First, ArUco tracking relies on line-of-sight visibility and sufficient lighting, making it sensitive to obstructions and lighting conditions. If a bird’s marker is obscured or the lighting is inadequate, tracking accuracy is compromised (Alarcón-Nieto et al., 2018; Eagan et al., 2022; Wolf et al., 2023). Unlike RFID, which functions even in complete darkness, ArUco tracking became ineffective after lights were turned off. This limitation prevented us from benchmarking perch use throughout the night. Instead, we were only able to monitor evening access and morning departure times, when lighting conditions allowed reliable detection.

Second, ArUco tracking primarily provides positional data but does not inherently capture directional movement. This limitation was particularly evident in nest box monitoring, where we could not confirm whether a hen remained inside, was perching, or was descending when it disappeared from detection. Consequently, human observation was occasionally required to resolve ambiguities, though this was minimal. To address this issue in future studies, researchers could incorporate orientation vectors or install cameras inside each nest box to automatically identify entry and exit behaviors. Each approach presents distinct challenges: reliable algorithm development for the former, and the need for additional equipment and data infrastructure (e.g., multiple cameras, storage) for the latter, which may pose logistical and funding constraints. When considering camera installation, care should be taken to ensure that devices do not compromise the internal space required by ethical guidelines, thereby avoiding disruption of the hens’ natural nesting behavior.

Understanding these limitations is essential for interpreting the results accurately and guiding future improvements in automated tracking systems.

## Conclusion

This study developed and automatically validated an RFID-based tracking system to monitor laying hens' interactions with functional resources in a controlled environment. The system reliably tracked several behaviors, including access to the wintergarden, night-time perching, and, to some extent, visits to the nest boxes. However, further refinement is needed to accurately capture interactions with the feeders and drinkers. These findings underscore the importance of rigorous validation for behavioral monitoring methods in poultry ethology, housing, and welfare research. Future improvements to RFID-based systems should involve a hybrid approach, combining hardware upgrades with advancements in software, as well as downstream data handling and analysis. Moreover, this study demonstrates the use of computer-assisted validation through 3D-ArUco tracking, which facilitates the evaluation of behavioral monitoring tools and supports the development and adoption of new tracking technologies in poultry science.

## Disclosures

The authors wish to confirm that there are no known conflicts of interest associated with this publication. The authors declare that the research was conducted in the absence of any commercial or financial relationships that could be construed as a potential conflict of interest.

## Appendix Supplementary materials

Image, application 1
