# Supplementary material for: Development and computer-assisted validation of a radio frequency identification system for tracking individual chicken visits to functional areas
Source: Poult Sci. 2025 Jul 31;104(10):105627. doi: 10.1016/j.psj.2025.105627 (PMC12355069; doi:10.1016/j.psj.2025.105627)
Supplement: Supplementary file 1 [file mmc1.docx]

**Supplementary Materials**

for the article:
**Development and Computer-Assisted Validation of a Radio Frequency Identification System for Tracking Individual Chicken Visits to Functional Areas**

Serge Alindekon, T. Bas Rodenburg, Jan Langbein, Birger Puppe, Olaf Wilmsmeier, Sebastian Wille, Helen Louton


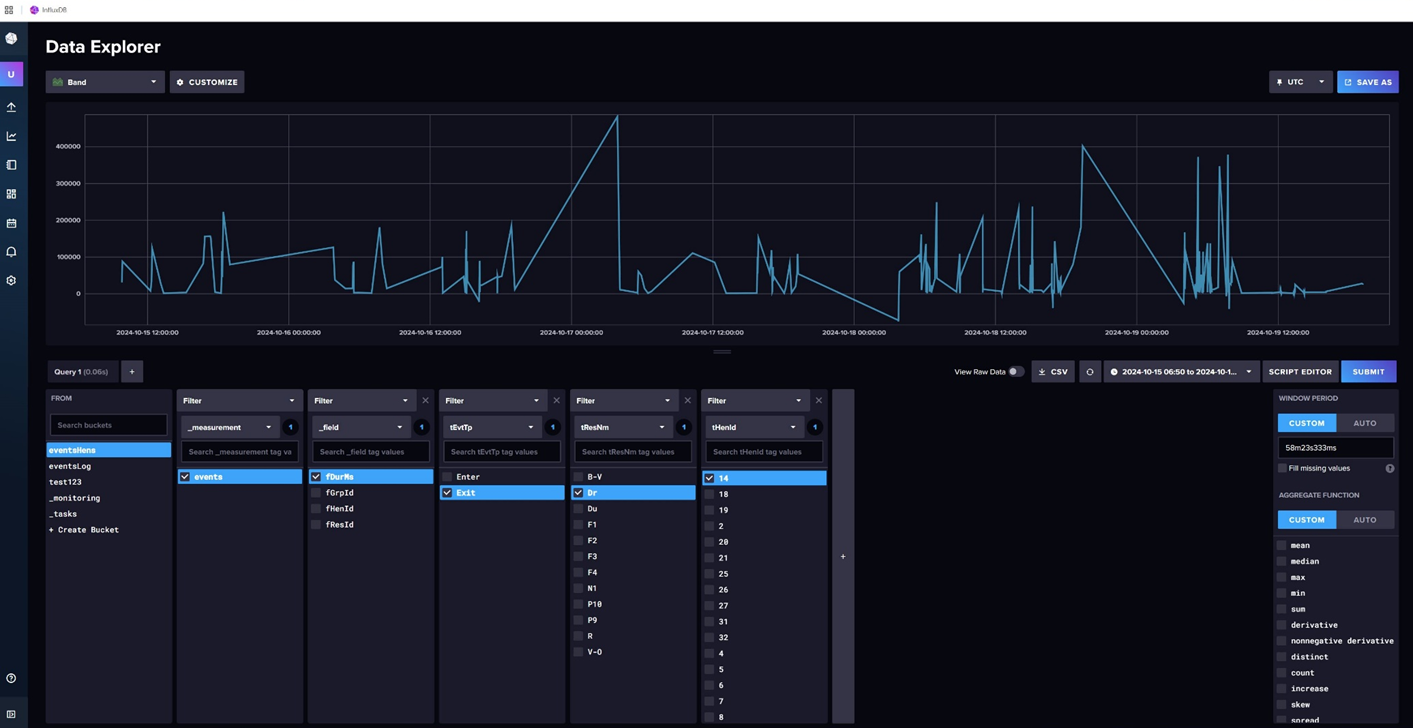


**Supplemental Material A:** Screenshot of the query interface in InfluxDB’s UI Data Explorer, showcasing a real-time time series visualization of RFID event data. The example displays visit durations for a single hen and a specific resource, with options to filter by RFID tag ID and timestamp range, based on automatically detected entry and exit events.


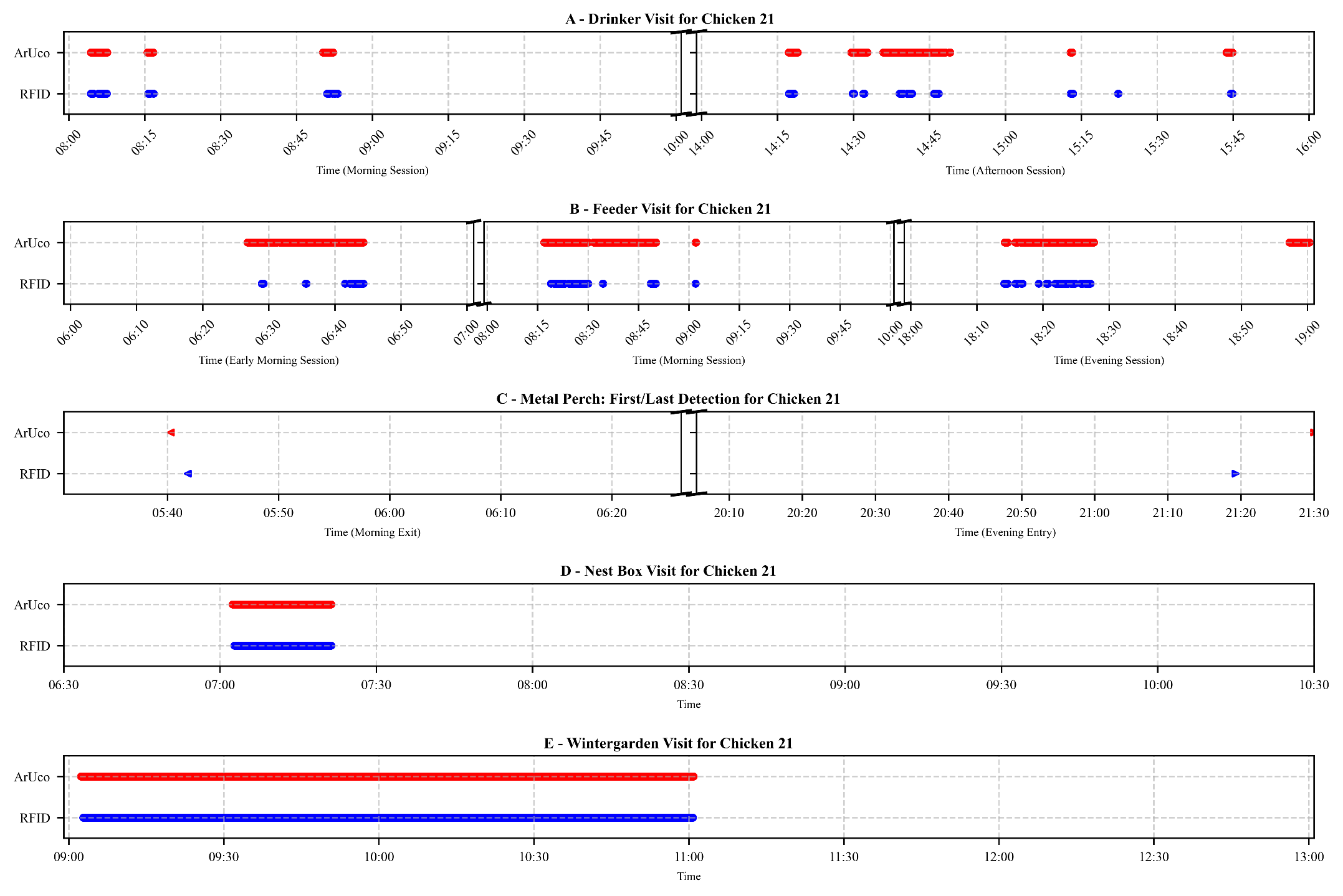


**Supplementary material B:** Visualization showing reconstructed visit events (post hoc) based on data recorded by the ArUco and RFID systems—Case of Chicken ID 21 on Day 3. This example was selected because the chicken was present across all resources on the same day, making it representative. Panels A and B respectively show the raw visit data for the feeder and drinker, without applying any bout criteria, based on the two detection methods. Panels D and E present the inferred visit data for the nest and wintergarden, where indirect inference approaches were applied. For these resources, blue lines (continuous or dashed) represent presence detected by RFID annotations, while red dots indicate ArUco detections, which serve as the gold standard. Panel C displays timestamps for access to and exit from the metal perch, according to both methods. Left-pointing arrows mark exit times, while right-pointing arrows indicate evening access. Red arrows represent ArUco annotations, and blue arrows correspond to RFID data.
